# The development of social learning: from pedagogical cues to selective learning

**DOI:** 10.3389/fpsyg.2024.1466618

**Published:** 2024-10-09

**Authors:** Mitsuhiko Ishikawa, Shoji Itakura

**Affiliations:** ^1^Hitotsubashi Institute for Advanced Study, Hitotsubashi University, Kunitachi, Japan; ^2^Research Organization of Open Innovation and Collaboration, Ritsumeikan University, Osaka, Japan

**Keywords:** social learning, development, pedagogical learning, selective learning, infants, children

## Abstract

Learning new information from others, called social learning, is one of the most fundamental types of learning from infancy. Developmental studies show that infants likely engage in social learning situations selectively and that social learning facilitates infant information processing. In this paper, we summarize how social learning functions support human learning from infancy focusing on two aspects of social learning; pedagogical learning and selective learning. We also provide an overview of the developmental process of social learning based on the findings of developmental research. This review suggests that the learning facilitation effects of pedagogical learning decrease with development, while the facilitation effects of selective learning are observed even in older ages. The differences in these learning facilitation effects are considered to be due to the differences in the utility of learning in uncertain environments. The findings of the studies imply the unique nature of human social learning and the critical role of social interactions in cognitive development. Understanding the development of social learning provides valuable insights into how infants learn and adapt in complex social environments.

## Introduction

Learning in the real world presents infants with complex challenges. As they develop, infants encounter a multitude of stimuli and potential learning opportunities, often without clear indications of what is important or relevant to learn. This ambiguity is particularly evident in word learning situations, where multiple potential referents exist for any heard word, making it difficult for infants to determine the meaning of an unknown word (Blythe et al., 2016). To navigate this complex learning environment and identify relevant information, infants engage in active exploration and information-seeking behaviors (Gottlieb et al., 2013). Since early studies of infant learning, literature has shown that infants tend to seek information from social sources and obtain new information mainly by interacting with and observing others (Baldwin and Moses, 1996). This type of learning in social situations is called social learning, which is the fundamental type of learning used across the animal kingdom to understand social life (Leadbeater, 2015). In contrast with other animals, because of social learning, human infants can acquire cultural knowledge from their social group during development (Tomasello and Carpenter, 2007). Thus, the literature has suggested that social learning leads to humans’ unique form of cumulative cultural evolution (Tennie et al., 2009). Social learning from infancy is crucial for knowledge transmission, which is unique in humans. In this paper, we provide an overview of how the social learning function supports human learning from infancy, focusing on the aspects of pedagogical learning and selective learning. Additionally, we summarize the developmental process of social learning based on the findings of previous developmental research.

## Pedagogical learning

Human communication is a fundamental way to transmit knowledge. Natural pedagogy theory proposes that communication enables fast, efficient social learning from early infancy (Csibra and Gergely, 2009). This theory argues that infants are sensitive to ostensive signals that indicate communicative intention. Empirical studies have shown that infants engage in social interaction and learning under situations with ostensive signals. For example, another’s direct gaze (eye contact) has been used as an ostensive signal in experimental settings. Newborns are sensitive to direct gazes toward them (Farroni et al., 2002). Other’s gaze information is a clue to learning about the environment. The literature has shown that pre-verbal infants use gaze information to refer to associations between words and objects (Baldwin, 1993). When a social partner says a word, infants refer to another’s gaze direction to identify the object associated with the word. Thus, gaze-following behavior is important for learning from early infancy. Furthermore, it has been reported that infants’ preference for and cognitive processing of a target are enhanced by following another’s gaze (Reid and Striano, 2005; Ishikawa and Itakura, 2018). This suggests that tracking others’ gaze is important for learning about the surrounding environment. The literature has demonstrated that eye contact facilitates gaze-following behavior in infants, suggesting that infants engage in more social interaction in situations with the ostensive signal than without it (Senju and Csibra, 2008; Ishikawa and Itakura, 2019; Ishikawa et al., 2022). Looking at the same object with another person has facilitative effects on infant information processing of the object after following another’s gaze direction (Okumura et al., 2013). Moreover, gaze following situation enhances infants’ preference for the face (Ishikawa et al., 2019). Gaze-following behavior in the early infancy predicts language development in later life (Brooks and Meltzoff, 2005, 2008; Okumura et al., 2017). Thus, facilitative effects of eye contact on infant gaze-following may provide learning opportunities for infants and have short-and long-term facilitative effects.

On the other hand, several studies suggest that the learning facilitation effect of ostensive cues may not be related to communicative intent, but rather may be a bottom-up effect caused by the attraction of attention. Szufnarowska et al. (2014) reported that infant gaze-following behavior was promoted by drawing their attention through facial movements, similarly to ostensive cues. Additionally, in the context of other action learning, it has been shown that motionese (child-directed action) can facilitate learning (Brand et al., 2002). Motionese refers to the use of exaggerated and repetitive hand gestures toward infants (Brand and Shallcross, 2008). Through these exaggerated movements, motionese captures infants’ attention to the action. These studies suggest that during infancy, bottom-up learning facilitation may occur more easily.

Another well-known ostensive signal is infant-directed speech (IDS). IDS is characterized by a higher and more variable pitch, shorter utterances and more vowel alterations than the speech style used to talk to adults (adult-directed speech: ADS) (Golinkoff et al., 2015). Along with eye contact, IDS facilitates infant gaze-following (Senju and Csibra, 2008; Okumura et al., 2020), and this facilitative effect was observed in people in Vanuatu regardless of culture and language (Hernik and Broesch, 2019). Moreover, IDS facilitates information processing after following another’s gaze direction (Yoon et al., 2008; Okumura et al., 2020). IDS has been investigated in word learning studies because of its benefits for speech processing (e.g., Háden et al., 2020; Zangl and Mills, 2007). Longitudinal studies have shown that frequent exposure to IDS increases vocabulary in later development (Ramírez-Esparza et al., 2014; Weisleder and Fernald, 2013). In experimental settings, IDS facilitates infants’ word segmentation (Schreiner and Mani, 2017; Thiessen et al., 2005) and their acquisition of word-object associations (Graf Estes and Hurley, 2013) more than ADS does. Studies have shown that IDS also facilitates infants’ learning.

Imitation has been observed in behavioral studies as an outcome of social learning (Over and Carpenter, 2013). Tomasello (1990) emphasises that imitation “involves the recognition and reproduction of the goal of the observed behavior, as well as the specific actions that brought about that goal.” In contrast, mimicry, another similar term, is defined by Tomasello et al. (1993) as “the replication of a model’s actions in the absence of any insight into why those actions are effective, or even what goal they served.” This distinction highlights the key difference: imitation involves understanding and reproducing both the action and its intended goal, while mimicry is merely a surface-level replication of observed actions without comprehension of their purpose. The importance of imitation in social learning is underscored by its crucial role in cultural transmission (Legare and Nielsen, 2015). Imitation enables the learning of complex behaviors that are difficult to explain verbally (Meltzoff, 1988), making it a powerful mechanism for transferring cultural knowledge and skills across generations. This goal-oriented nature of imitation not only facilitates more efficient and adaptive learning but also potentially contributes to the development of social cognition and understanding of others’ intentions.

Ostensive signals also facilitate imitations. Real interaction studies have shown that calling a child’s name and grabbing her attention before the action enhanced the child’s imitations after the observation (Király et al., 2013; Southgate et al., 2009). Moreover, eye contact facilitates mimicry of intransitive hand movements (Wang et al., 2011) and facial mimicry (de Klerk et al., 2018). The facilitative effects of ostensive signals on imitation have been shown in various age groups. For example, 8-to 13-month-old infants are more likely to use a tool successfully when they observe other’s tool use with ostensive communication (Sage and Baldwin, 2011). Moreover, 15-to 18-month-old infants are more likely to imitate the actions of an actor who ostensibly demonstrates action than an actor who addresses no one (Brugger et al., 2007; Matheson et al., 2013). Recent research on imitation has shown that ostensive signals not only convey communicative intentions but also alert infants to when they should focus their attention for learning. Kliesch et al. (2022) found that using ostensive signals as segmentation cues in action sequence learning enhanced imitation in 18-month-olds. Children may use ostensive signals not only to expect communication but also to segment incoming events or action sequences. Thus, ostensive signals serve as cues for when to engage in social learning.

## Selective learning

Deciding who to learn from is important for social learning in uncertain situations. Studies on selective learning have shown that infants can select informants who are accurate (Koenig and Sabbagh, 2013). The reliability of informants is essential to deciding who to learn from. In this context, the reliability of informants refers to individuals who provide statistically reliable information and/or who do not offer information irrelevant to the task or action goal. Reliable informants deliver accurate and pertinent information that aligns with the objectives of the task or action at hand. The reliability of informants has been examined in experimental tasks through the manipulation of observational consistency and behavioral predictability. The literature has shown that approximately 8-month-olds infants can track the reliability of informants (Tummeltshammer et al., 2014). Tummeltshammer et al. (2014) presented faces cueing the locations of animations with different levels of reliability (i.e., reliable: always directed her gaze toward the box where the animal would appear; unreliable: Directed her gaze toward the box where the animal would appear on only one out of every four trials); they found that 8-month-old infants looked longer at the locations cued by the reliable faces than at those cued by the unreliable faces. In this study, reliability was manipulated based on the probability that gaze direction predicted an event (animal appearing), but it is possible that children built expectations about others’ gaze information from their experiences. In this interpretation, reliability can be viewed as the informativeness or goal-directedness of others’ gaze. By repeatedly observing the gaze cueing situation, children may learn the goal-directedness of others’ gaze toward where the event occurs, creating an expectation for the informativeness of other’s gaze. Other studies have also shown that children engage in selective social learning more with reliable informants than with unreliable ones (Koenig and Harris, 2005; Koenig and Sabbagh, 2013; Mills, 2013; Nurmsoo et al., 2010). For example, Chow et al. (2008) compared gaze-following behavior between reliable and unreliable conditions in infants aged 14–16 months. Under the reliable condition, infants observed an actor that showed positive expressions while looking inside a container with a toy inside. Under the unreliable condition, infants observed an actor that showed positive expressions while looking inside an empty container. After observing these situations, the infants conducted gaze-following tasks with the actors. The infants showed more frequent gaze-following in the reliable condition than in the unreliable condition, suggesting they selectively engaged in social learning with the reliable informant. The same manipulation of informants’ reliability was used in a study on selective imitation. In the imitation study, infants watched as an actor turned on a touch-light using her forehead (Poulin-Dubois et al., 2011). After this observation, the infants were given time to attempt to turn on the light. The results showed that infants in the unreliable condition were more likely to use their hands than their foreheads to turn on the light and that infants in the reliable group were more likely to imitate the actor’s action by using their foreheads. Thus, as shown in the studies of gaze-following and imitation, the reliability of informants modulates selective social learning in infants. In the context of cultural learning, children’s ability to assess the accuracy of information sources appears to be a crucial cognitive mechanism. This capacity extends beyond the familiar-unfamiliar dichotomy, suggesting that children evaluate the reliability of information independently of their relationship with the informant. Research indicates that when presented with conflicting sources, children prioritize demonstrated accuracy over long-standing familiarity (Corriveau and Harris, 2009). This finding underscores the sophisticated nature of children’s information-gathering strategies in cultural learning contexts, highlighting their ability to discern and prioritize reliable sources of knowledge regardless of pre-existing relationships.

Social group membership has also been manipulated in infant selective learning studies. For example, linguistic membership is a clue to distinguishing the in-group and out-group for infants. Infants from 5 to 6 months old showed a looking preference for a person who previously spoke their native language (Kinzler et al., 2007), and 7-month-olds listened longer to a tune that was introduced by a native speaker than by a foreign speaker (Soley and Sebastián-Gallés, 2015). These studies suggest that infants aged approximately 6 months have visual and auditory preferences for their native speakers (in-group), and such preferences may contribute to selective learning. In addition, infants aged approximately 18 months have been shown to imitate the in-group actor more than the out-group actor (Howard et al., 2015; Altınok et al., 2022). Buttelmann et al. (2013) found that 14-month-old infants imitated the in-group actor more than the out-group model, suggesting that at this age, cultural learning has already begun.

### Development of social learning

From the neonatal period, babies have high sensitivities to social stimuli, such as attentional bias to another’s direct gaze (Farroni et al., 2002). Fetuses were shown to have an attentional bias to a top-heavy, face-like stimulus (Reid et al., 2017). This primitive attentional preference may be operated by configuration-sensitive mechanisms. Farroni and colleagues (Farroni et al., 2005) demonstrated that newborns’ preference for faces with a direct gaze is observed only within the context of an upright face and a straight head, suggesting that the driver of primitive preferences for faces may be the configuration of upright faces. Initial perceptual bias towards faces may provide opportunities to engage in social stimuli and learn about the environment in development (Johnson et al., 2015). Thus, infants possibly engage in social learning from the early stage of life.

Studies have shown that social learning within the context of pedagogical and selective learning is facilitated in infants before they turn 18 months old. However, these facilitative effects may be decreased or vanish in later development. For example, 20-month-old infants equally follow another’s gaze direction in situations with or without eye contact (de Bordes et al., 2013), suggesting that the effects of ostensive signals are decreased at this age. Matheson and colleagues (Matheson et al., 2013) compared imitation between 18-and 24-month-old infants in social learning situations with or without interaction. They found that 18-month-old infants showed more imitations in the interactive situations than the 24-month-old infants, and the 18-and 24-month-old infants equally imitated the actor’s action between interactive and noninteractive situations. These studies suggest that the facilitation effects of pedagogical learning may decrease during development. Computational modeling of the learning process of infant gaze-following behavior suggests that the facilitative effects of ostensive signals on gaze-following decrease during development because infants learn the action value of gaze-following regardless of the presence of ostensive signals (Ishikawa et al., 2020). A longitudinal study revealed that infants’ gaze following develops with the linear increasing pattern of age-related growth between 9 and 18 months (Mundy et al., 2007). The learning of action utilities may progress in socially engaging actions. As a result, paying attention to and engaging with another person is rewarding regardless of the communicative intent of others (Ishikawa and Itakura, 2022). Ishikawa and colleagues (Ishikawa et al., 2021) showed that direct gaze did not affect attentional shifts to another’s gaze direction, suggesting that eye contact did not affect gaze-following in adults. In early development, because what should be paid attention to and learned is uncertain, ostensive signals guide learning behavior, enhancing infants’ social engagement such as gaze following. However, if the value or utility of social actions is learned in the development, humans may be motivated to engage in social situations regardless of ostensive signals. Also, the development of attention during social interaction undergoes significant changes in infancy. It’s important to consider the top-down influences on social interaction in development. For example, Rohlfing and Nomikou (2014) observed that at three months, infants predominantly focus their attention on their mothers during interactions. By six months, however, infants’ attention becomes more distributed, indicating a shift from a singular focus on the mother to a broader attentional scope. This developmental shift suggests that infants are acquiring the ability to anticipate social sequences, an indication of emerging top-down cognitive processes, whereby infants begin to expect certain action sequences based on prior experiences. As these top-down influences strengthen, they may progressively diminish the salience of ostensive signals—bottom-up cues that initially play a critical role in signaling learning opportunities within social contexts.

By contrast, selective learning is beneficial in older ages. Burdett and colleagues (Burdett et al., 2016) compared the selective learning of instrumental tool use in situations with a competent or incompetent actor. The result showed that children aged between 4 and 7 years prefer to copy the competent actor, suggesting that children selectively learn from experts. Sobel and Finiasz (2020) conducted a meta-analysis of children’s selective word learning, reviewing 63 papers on 6,525 participants. Although the children’s task performances depend on age and task types, selective learning of words can be observed in children aged between 2 and 5 years. Oláh and Király (2019) compared the selective learning of instrumental tool use in 3-year-olds after they watched videos depicting two models: one who performed conventional tool-using actions, and one whose tool-using actions deviated from social conventions. Moreover, they used two demonstration situations with or without ostensive signals. In this study, children were more likely to copy the actions of the conventional actor, regardless of the ostensive signals. Thus, the effects of ostensive signals on social learning may decrease during development, and children continue to engage in selective learning. Selective learning retains its benefit in later development because it is supported by the knowledge or experience of others for efficient learning, top-down modulations on learning. Although social interaction can be rewarding during development, learning from an unreliable or out-group informant can cause non-rewarding or threatening situations. For example, out-group faces are recognized as threatening or fearful stimuli, signaling untrustworthy information (Mallan et al., 2013). An argument is that learning from out-group members could be less efficient or counterproductive and that selective learning could have evolved because it minimized the risk of deception (Montrey and Shultz, 2022). Gaze-following studies in adults have also suggested that top-down social information modulates social engagement. Top-down modulations, such as effects of group membership (Ciardo et al., 2014) and social status (Dalmaso et al., 2012), have been demonstrated to affect adults’ gaze-following. Thus, knowledge-based top-down modulations may have advantages in social engagement and learning in later development.

Another reason why selective learning continues to have a strong influence later in development could be due to infants gradually becoming members of a social group as they develop. As known in the phenomenon of perceptual narrowing (Werker and Tees, 1984), infants possess the ability to adapt to various environments early in development, but as they grow up, they begin to engage in cognitive processing that is tailored to their specific environment. For example, it has been shown that racial membership strengthens with development (Pauker et al., 2016). As infants develop in becoming members of a social group, the importance of learning from others within the same social group may also increase. Thus, it can be considered that selective learning is prioritized through adaptation to the developmental environment.

### Future direction

As outlined above, social learning guides infants on when and what to learn in the early stages of development. The question of what aspects make social learning a unique learning style remains unresolved. For example, studies of correlations between domain-general learning abilities and infants’ selective social learning have shown no links, suggesting that social learning cannot be based on domain-general learning (Crivello et al., 2018; Crivello and Poulin-Dubois, 2019; Luchkina et al., 2018). A recent study also found no correlation between selective social learning and associative learning skills (Crivello et al., 2021). These studies insist that social learning is not a mere extension of associative learning in social situations. A recent study demonstrated that 18-month-old infants are more inclined to learn from an informant under uncertain situations despite the informant being unreliable, suggesting that the uncertainty of the environment modulates social learning (Kuzyk et al., 2020). Thus, the information-seeking tendency under uncertainty is correlated with social learning abilities. Further research is needed to examine how social learning differs from other forms of learning.

Also, future research should further investigate the mechanisms underlying the decrease in the effects of ostensive signals as infants grow older. Longitudinal studies could provide insights into how infants’ learning strategies evolve and how the facilitative effects of pedagogical and selective learning change over time.
